# CAV-1 Overexpression Exacerbates the Manifestation in EPAC-1-Induced Chronic Postsurgical Pain in Rats

**DOI:** 10.1155/2022/8566840

**Published:** 2022-07-31

**Authors:** Qian Hua, Shiren Shen, Yibin Qin, Su Cao

**Affiliations:** ^1^Medical College, Nantong University, Nantong, Jiangsu 226001, China; ^2^Department of Anesthesiology, Affiliated Hospital of Nantong University, Nantong University, Nantong, Jiangsu 226001, China

## Abstract

**Purpose:**

Caveolae (CAV) are an invaginated microcapsule with the shape of Ω on the surface of the cell membrane. Caveolin-1 (CAV-1) is involved in neuropathic pain, and adenosine monophosphate (AMP)-exchange protein directly activated by cAMP1 (EPAC-1) is a potential therapeutic target for chronic pain. However, whether EPAC-1 promotes chronic postsurgical pain (CPSP) through CAV-1 has not been reported. Here, we aim to investigate the underlying mechanism of CAV in CPSP.

**Methods:**

All the rats were divided into 9 groups, including the Naive group, Sham group, skin/muscle incision and retraction (SMIR) group, SMIR + CAV-1 siRNA group, SMIR + control siRNA group, SMIR (7 days)+Saline group, SMIR (7 days)+CE3F4 group, 8-PCPT group, and Saline group. The CPSP rat model was established after SMIR. A mechanical withdrawal threshold (MWT) was recorded to evaluate the animal's behavior. Western blotting and immunofluorescent were performed to detect the protein expression levels of EPAC-1 and P-CAV-1.

**Results:**

EPAC-1 and CAV-1 were both overexpressed after operation, particularly in astrocytes, microglia, and neurons of spinal marrow (all *P* < 0.05). Interestingly, CAV-1 siRNA can partly reverse the SMIR-induced hypersensitivity, but there was no effect on EPAC-1. Besides, EPAC-1 blockage partly reversed the SMIR-induced hypersensitivity and CAV-1 overexpression, and EPAC-1 activation promoted CAV-1 overexpression and hypersensitivity in normal rats (all *P* < 0.05).

**Conclusion:**

CAV-1 mediates the functional coupling of microglia, astrocytes, and neurons, and thus EPAC-1/CAV-1 plays an important role in CPSP exacerbation.

## 1. Introduction

Chronic postsurgical pain (CPSP) is defined as the surgical-related pain in or around the operation lesion, lasting more than 2 months. Appropriate 10–50% of patients have suffered CPSP after common surgeries, for instance, inguinal hernia repair, radical mastectomy, and cesarean section, which severely impacts the prognosis and life quality of patients after surgery. Till now, no effective approach could effectively prevent or treat CPSP. Therefore, it is imperative to elucidate the underlying mechanism of CPSP.

Similar to the classical protein kinase A (PKA) signaling pathway, cyclic adenosine monophosphate (cAMP) can modulate multiple physiological and pathophysiological processes, independent of PKA, via the exchange protein (EPAC) [1–4]. The cAMP/PKA signaling is localized in the fossae (Caveolae, CAV) and mediated by caveolin-1 (Caveolin-1, CAV-1), which has roles in lipid metabolism, dendritic growth, and pathogenesis-related to ischemic injury [5–8]. CAV is an invaginated microcapsule with the shape of Ω on the surface of cell membranes, where a variety of signal transduction pathways converge. CAV-1 is a mosaic protein that modifies the inner surface of CAV, participating in cell signal transduction, cell membrane interactions, and intracellular environmental balance. Spinal cord microglia P-CAV-1 is reported to be persistently upregulated in neuropathically injured rats, promoting mechanical hyperalgesia [9–12]. The cAMP-EPAC-1 signal plays a key role in chronic inflammatory pain, considered a potential therapeutic target for chronic pain [13–15]. However, the role of CAV-1 in promoting chronic inflammatory pain via EPAC-1 has not been fully understood.

Skin/muscle incision and retraction (SMIR) is a rat model of postsurgical pain through pulling on the skin and muscle tissue for an extended duration of time without causing damage to the main peripheral nerves around the incision. SMIR can mimic the features of an inflammatory microenvironment and the CPSP process in the operative area, which has been widely applied in CPSP studies [16].

In this study, a CPSP model of SMIR rats was established to observe the postoperative alterations and distribution of EPAC-1 and p-CAV-1 in the spinal marrow, as well as the relationship between them, exploring the role of CAV-1 in EPAC-1-induced CPSP.

## 2. Material and Methods

### 2.1. Animals

54 male Sprague Dawley rats, aged 8–10 weeks and weighing 200–250 g, were purchased from the experimental animal center of Nantong University (Nantong, China). They were maintained in a 12 h light/dark cycle at 23 ± 1˚C with 55–60% humidity and given free access to food and water until 3 days before the start of the experiment. All animal protocols received approval from the Animal Care and Use Committee of Nantong University (approval no. 20171015S1051122; Nantong, China).

### 2.2. Experimental Design and Drugs

**Figure 1:**
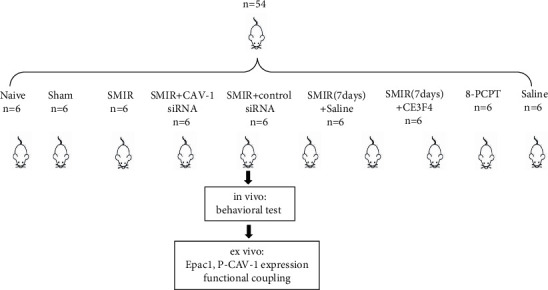
The graphical timeline of experimental protocols in this study.

54 rats were randomly and evenly divided into 9 groups, and the detailed grouping information was listed as follows: (1) Naive group: in the naive group, no treatment was performed. (2) Sham group: rats were injected intraperitoneally with 0.3% pentobarbital sodium 30 mg/kg for anesthesia and placed in a supine position. Next, 3–4 mm inside the saphenous vein in the middle thigh of the right hind limb, the skin was cut 1.5–2 cm under aseptic conditions and sutured, and the incision was covered with aureomycin ointment. (3) SMIR group: based on the sham group, the superficial muscles were exposed and bluntly separated at 7 mm–10 mm, according to methods like Flattes et al. [16], and the retractor clasp was put in and propped up to 2 cm for 60 minutes before suturing. (4) SMIR + CAV-1 siRNA group: 10 nmol CAV-1 siRNA (RiboBio, China) was injected into the sheath at the same time point of control siRNA. (5) SMIR + control siRNA group: 10 nmol of control siRNA (RiboBio, China) was injected into the sheath 1 day before and 1 day after SMIR modeling. (6) SMIR (7 days) +Saline: 7 days after SMIR modeling, the right plantar of the rat was injected with the same dose of normal saline. (7) SMIR (7 days)+CE3F4 group: 7 days after SMIR modeling, the right plantar of rats was injected with EPAC-1 inhibitor CE3F4 (Tocris Company, UK) 1ug. (8) 8-PCPT group: normal rats were injected with EPAC-1 agonist 8-PCPT (Absin Bioscience Inc., China) 1ug on the right plantar. (9) Saline group: the same dose of normal saline was injected at the same site as the SMIR + CE3F4 group. The graphical timeline of experimental protocols is shown in Figure 1. During the study, we had different divisions of labor. The operators of breeding and surgical operations are independent of the experimental performance operators, thus guaranteeing the reliability of results.

### 2.3. Behavioral Testing

Based on the protocol reported previously [16], the mechanical withdrawal threshold (MWT) was recorded at 1, 3, 7, 14, and 28 preoperative and postoperative days. The rats were adapted to stand on the metal screen of the organic glass box (22 × 12 × 22 cm³) for 30 min. Under resting conditions, the hind paws of rats were stimulated by the von Frey filament cilia stimulator (North Coast Medical, USA). The logarithmically shifted stiffness was 1.4 g–26 g. The Von Frey was slightly angled to produce the standard total force and the stimulation period was adjusted to ≤4 s [16]. For our assessment purposes, paw withdrawal, paw licking, and other such behaviors were marked as positive, whereas the absence of response was deemed as negative. Our experiments started with 1.4 g, and the endpoint was determined by the lifting method. Each experiment was conducted 5 times with an interval of 30 s. Response in ≥2 instances out of 5 was regarded as mechanical touch-induced pain. If the number of responses was <2, then the following upper-level stimulation was employed. In the case of ≥2 positive responses, the stimulation strength was lowered by one level. This was continued until a positive or negative reaction straddle was achieved. The paw withdrawal threshold was determined based on the threshold table and the unit applied was “g.” In terms of mechanical hyperalgesia, the lower the paw withdrawal threshold, the greater the degree of mechanical hyperalgesia.

### 2.4. Western Blotting

The rats were anesthetized and sacrificed, and L4-5 spinal lumbar enlargement was collected and stored at −80°C. After thawing on ice, 1 mL of RIPA cracking solution was added per 100 mg of tissue for 10 min, and the homogenate was electrically homogenated for 30 min before transfer into an EP tube, followed by centrifugation at 4°C at 10000 × *g* for 15 min, and then the supernatant was divided into 100 *μ*L per tube. The protein concentration was identified by the BCA assay. The protein samples (30 *μ*g) were separated by SDS-PAGE (Bio-Rad, USA) and transferred to a 0.2 *μ*m PVDF membrane. Then sealing was done with a 5% BSA solution at room temperature (RT) for 2 h, and then the membrane was incubated with primary antibodies (in blocking buffer) against the following proteins overnight (O/N) at 4°C: EPAC-1(1 : 50, Santa, USA), P-CAV-1(1 : 50, Santa, USA), and *β*-actin (1 : 5000, Sigma, USA). The next day, the membrane was incubated with the corresponding secondary antibody (1 : 5000, Jackson, USA) at RT for 2 h. The ECL chemiluminescence detection kit (Absin, China) was applied to visualize the membrane using the Tanon 2500 gel imaging system (Yph-Bio Co., Ltd., Beijing, China). Protein band quantification was done with Image J (National Institutes of Health, Bethesda, MD, USA) and *β*-actin was employed as an endogenous control.

### 2.5. Immunofluorescence

Briefly, saline was flushed through the heart to remove blood components. Then fixation was performed via perfusion of 250 ml of 4% paraformaldehyde. Next, the heart was excised and dehydrated sequentially in 20% and 30% sucrose solution, frozen, and sliced to a thickness of 6 *μ*m. During immune staining, a specified number of slices were arbitrarily picked, rinsed in 0.01 mol/L PBS, and blocked with a 5% serum antibody blocking solution for 2 h at RT. Primary antibody dilutions were done with a 1% serum antibody dilution. The sliced specimens were then incubated O/N at 4°C with one of the following antibodies: Epac1 (1 : 50, Santa, USA), p-cav-1 (1 : 50, Santa, USA), NeuN (1 : 500, Cell Signaling Technology), GFAP (1 : 500, Cell Signaling Technology), and Iba1 (1 : 500, Wako), before 3 rinses with 0.01 mol/L PBS for 10 min, followed by incubation with subsequent secondary antibody in the dark for 2 hours at RT. Secondary antibody dilutions were performed in 0.01 mol/L PBS: Cy3-goat anti-rabbit IgG (1 : 1000, Jackson, USA) and FITC-goat anti-mouse IgG (1 : 1000, Jackson, USA). Following 3 15-min rinses with 0.01 mol/L PBS, the specimens were then mounted in the dark and the films were prepared under a fluorescence microscope (OLYMPUS, Japan).

### 2.6. Intrathecal Injection of siRNA

CAV-1 siRNA was designed and synthesized by RiboBio Co., LTD. Cav 1 siRNA sequence: 5′-CCAGAAGGGACACACAGTT-3′. Control siRNA sequence: 5′-GAGAAGCAGUGAUACGACG-3'. The rats were anesthetized and placed in the supine position, and the surrounding hair was shaved. Under aseptic conditions, the rats were punctured between the spinous processes of L5-6 with a microinjector, and the flutter or sudden lateral swaying of the tail was a sign of successful puncture of the subarachnoid space. After the needle was fixed, CAV-1 siRNA (20 *μ*L) or the equivalent volume of control siRNA was injected.

### 2.7. Data and Statistical Analyses

The SPSS 20.0 software (SPSS Inc., Chicago, IL, USA) was used for the data analysis. GraphPad PRISM software version 6 (GraphPad Software, San Diego, CA, USA) was applied for plotting statistical graphs. Behavioral data analysis was done with two-way analysis of variance (ANOVA) with the subsequent Bonferroni test, which served as the multiple comparison analysis. Image J (National Institutes of Health, USA) was employed for the immunofluorescent analysis, and one-way analysis was used for the comparison among three or more groups for western blot. Unpaired *t*-test compared between the two groups. *P* < 0.05 was set as the significance threshold.

## 3. Results

### 3.1. Establishment of SMIR Model in Rats and Detection of MWT

**Figure 2:**
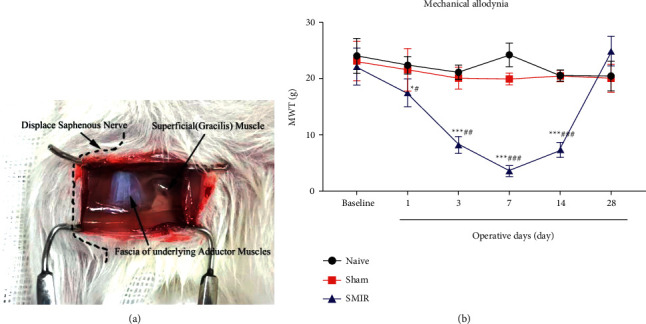
(a)The dotted line represents the saphenous nerve and the deep adductor fascia in white. (b) MWT of the SMIR group was significantly reduced time-dependently on the 1st, 3rd, 7th, and 14th days after the operation; however, the 14th day after the operation began to grow up. *n* = 6. ^*∗*^*P* < 0.05, ^*∗∗∗*^*P* < 0.001, compared with the base value; #*P* < 0.05, #*P* < 0.01, ###*P* < 0.001, compared with the Naive or Sham groups.

We established the CPSP model of SMIR rats based on the study of Flattes et al. [16] (A). Von Frey filament cilia stimulator was used to determine the ipsilateral claw base threshold (B). Compared with the Naive or basal values, the MWT of SMIR group was significantly reduced on the 1st, 3rd, 7th, and 14th days after the operation (*P*=0.031, *P*=0.001). The test failed to detect a difference in the sham group (*P*=0.061), indicating that the CPSP model of SMIR rats was successfully prepared (Figure 2).

### 3.2. Expression and Cellular Localization of P-CAV-1 Protein in the Spinal Marrow of SMIR Rats

**Figure 3:**
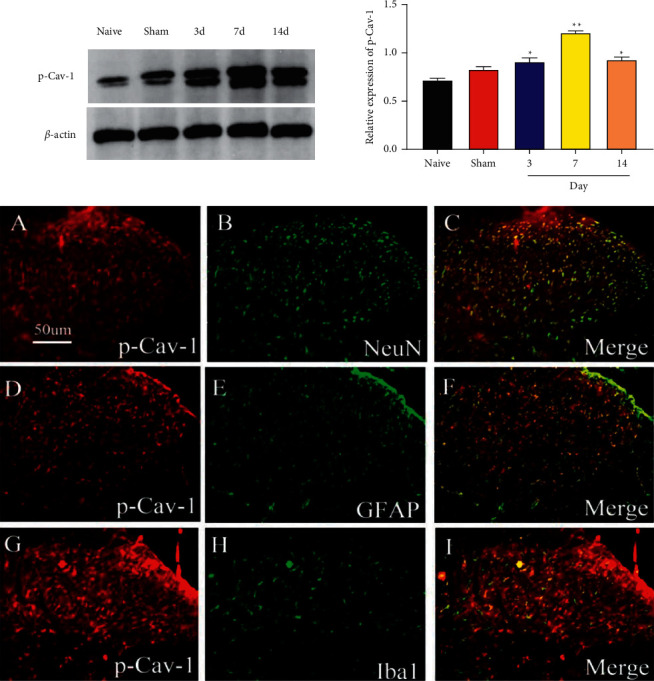
Expression and localization of CAV-1 protein in the spinal marrow of SMIR rats. Relative to the Naive rats, the SMIR rats showed significant increases in p-CAV-1 expression level (red fluorescence) on the 3rd (a), 7th (d), and 14th (g) days after the operation (*P* < 0.05, *P* < 0.01), whereas no obvious differences were observed in the Sham rats. Besides, p-CAV-1 was costained with NeuN (b), GFAP (e), and Iba1 (h) (green fluorescence). The merged images were presented in (c), (f), and (i) *n* = 6. ^*∗*^*P* < 0.05; ^*∗∗*^*P* < 0.01; ^*∗∗∗*^*P* < 0.001, compared with the Naive group.

Spinal cord tissues were selected on the 1st, 3rd, 7th, and 14th days after the operation, and P-CAV-1 protein expression was detected via Western Blot. Spinal cord tissue sections were prepared on the 7th day after surgery, and p-CAV-1 was costained with neuronal (NeuN), astrocyte (GFAP), and microglia (IBA1) markers using double-label immunofluorescence staining. Our data revealed that, relative to the Naive rats, the SMIR rats showed significant increases in p-CAV-1 expression levels on the 3rd, 7th, and 14th days after the operation (*P*=0.024*P*=0.002), whereas no obvious differences were observed in the Sham rats (*P*=0.087). This evidence indicated that CAV-1 expression was inactivated after surgery, and astrocytes, microglia, and neurons were the anatomical basis for the CAV-1-mediated regulation of CPSP pain information (Figure 3).

### 3.3. Expression and Cell Localization of Epac-1 Protein in the Spinal Marrow of SMIR Rats

**Figure 4:**
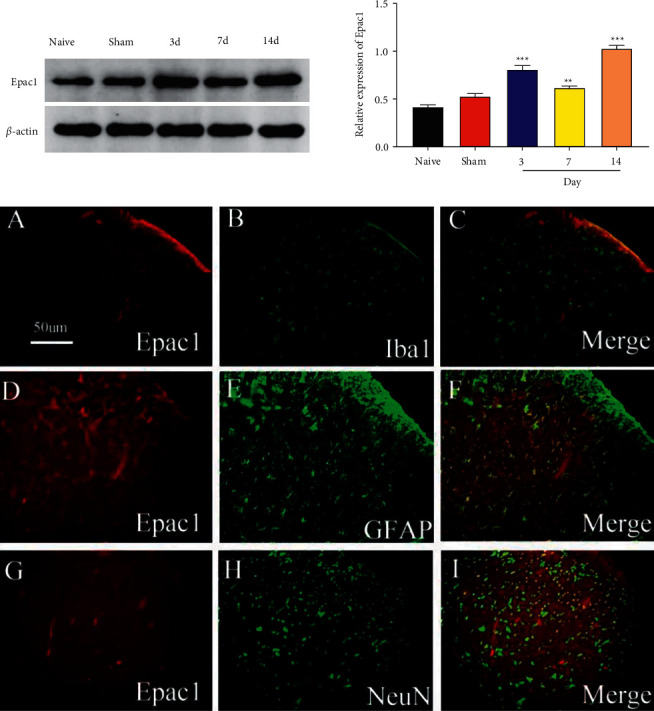
Relative to the Naive rats, the SMIR rats showed significant increases on the 3rd (a), 7th (d), and 14th (g) days after the operation (*P* < 0.01, *P* < 0.001), whereas no obvious difference was observed in the Sham rats. The staining was colabeled with GFAP (e), but not colabeled with Iba1(b) and NeuN (h). The merged images were presented in (c), (f), and (i) *n* = 6. ^*∗∗*^*P* < 0.01; ^*∗∗∗*^*P* < 0.001, compared with the Naive group.

The spinal cord tissues were collected on the 3rd, 7th, and 14th days after the operation, and the expression of Epac-1 protein was detected via Western Blot. Spinal cord tissue sections were prepared on day 7 after surgery, and Epac-1 was costained with NeuN, GFAP, and Iba1 using double immunofluorescence staining. It is shown that Epac-1 was significantly upregulated on the 3rd, 7th, and 14th days after the operation compared to Naive rats (*P*=0.004, *P*=0.001), no obvious difference was found in the Sham rats. The staining was colabeled with GFAP but not colabeled with Iba1 and NeuN. The results indicated that Epac-1 expression was inactivated after operation, and astrocytes were the anatomical basis for its regulation of CPSP pain information (Figure 4).

### 3.4. Effects of CAV-1 siRNA on Pain Sensitivity and Spinal Marrow Expression of p-CAV-1 and Epac-1 in SMIR Rats

**Figure 5:**
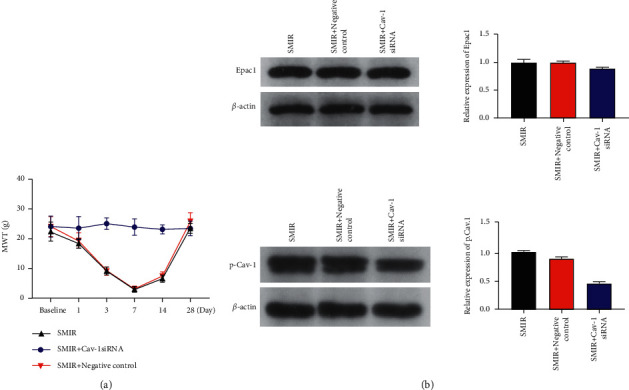
(a) Relative to the rats in SMIR and the SMIR + negative control group, the MWT of the SMIR + Cav-1 siRNA rats increased significantly at 1, 3, 7, and 14 days after injection. (b) Compared with the SMIR rats 7 days after surgery, no obvious difference was observed in Epac-1 in the SMIR + CAV-1 siRNA rats. However, p-CAV-1 was drastically reduced (*P* < 0.01), and the SMIR + control siRNA-treated rats showed no significant change (c). *n* = 6. ^*∗*^*P* < 0.05, ^*∗∗*^*P* < 0.01; ^*∗∗∗*^*P* < 0.001, compared with the SMIR group.

CAV-1 siRNA or control siRNA was intrathecally injected 1 day before and 1 day after surgery. Spinal cord tissues were selected on the 7th day after surgery. The results showed that the MWT increased significantly in the SMIR + CAV-1 siRNA-treated rats, compared to the SMIR rats on 1, 3, 7, and 14 postoperative days (*P*=0.032, *P*=0.001). However, no significant difference was found in the SMIR + siRNA-treated rats. Our findings indicated that CAV-1 activation significantly increased pain sensitivity, and Epac-1 may be the upstream regulatory mechanism of CAV-1 (Figure 5).

### 3.5. Effects of CE3F4 on Pain Sensitivity and Spinal Marrow p-CAV-1 Expression in SMIR Rats

**Figure 6:**
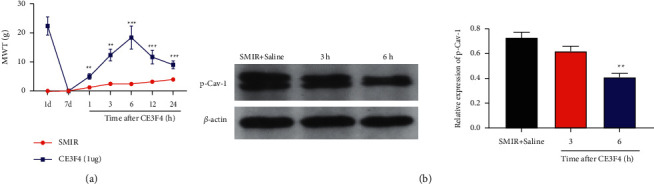
(a) Relative to the SMIR rats, the MWT of the SMIR + CE3F4 rats increased significantly at 1, 3, 6, 12, and 24 h after injection (*P* < 0.01, *P* < 0.001). (b) P-CAV-1 was significantly decreased at 3 h and 6 h after injection (*P* < 0.01). (^*∗*^*P* < 0.05, ^*∗∗*^*P* < 0.01; ^*∗∗∗*^*P* < 0.001, compared with the SMIR group). *n* = 6.

Seven days after SMIR, the rats were injected with CE3F4, the Epac-1 inhibitor, or the same volume of normal saline (SMIR group). It is shown that the MWT of the SMIR + CE3F4 rats increased significantly at 1, 3, 6, 12, and 24 h after injection (*P*=0.003, *P*=0.001), compared to SMIR rats (A). P-CAV-1 was significantly decreased at 3 h and 6 h after injection (*P* < 0.01) (B). These results indicated that the activation of Epac-1 by SMIR significantly increased pain sensitivity via upregulation of p-CAV-1 (Figure 6).

### 3.6. Effects of 8-PCPT on Pain Sensitivity and Spinal Marrow p-CAV-1 Expression in Normal Rats

**Figure 7:**
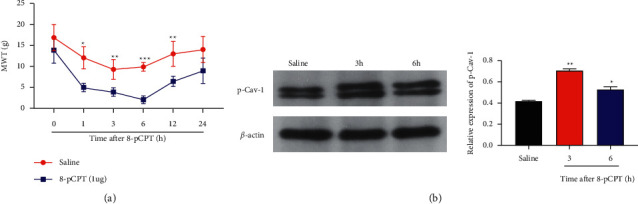
(a) Relative to the Saline rats, the MWT of the 8-PCPT group was significantly decreased at 1, 3, 6, and 12 h after injection (*P* < 0.05, *P* < 0.01, *P* < 0.001). (b) P-CAV-1 was significantly increased at 3 h and 6 h after injection (*P* < 0.05, *P* < 0.01). (^*∗*^*P* < 0.05; ^*∗∗*^*P* < 0.01; ^*∗∗∗*^*P* < 0.001, compared with the SMIR group) (*n* = 6).

Normal rats were injected with 8-PCPT, an Epac-1 agonist, or the same volume of normal saline (Saline rats). The results showed that compared to the Saline rats, the MWT of the 8-PCPT group was significantly decreased at 1, 3, 6, and 12 h after injection (*P*=0.021, *P*=0.003, *P*=0.001) (A), and P-CAV-1 was significantly upregulated at 3 h and 6 h after injection (*P* < 0.05, *P* < 0.01) (B), suggesting that Epac-1 activation significantly increases pain sensitivity by upregulating p-CAV-1 (Figure 7).

## 4. Discussion

Pain related to postoperation for more than 2 months is considered CPSP, affecting 10–50% of patients. In the current study, we identified that CAV, an invaginated microcapsule with the shape of Ω on the surface of the cell membrane, can mediate the functional coupling of the microglia-astrocyte with neurons. In the process, EPAC-1/CAV-1 acts as an important signaling pathway to exacerbate the manifestations of CPSP.

SMIR induces the activation of spinal microglia and astrocytes and upregulates TNF-*α* and other inflammatory mediators to promote CPSP [17, 18]. In the SMIR model, microglia in rats induced the transformation of spinal astrocytes to the A1 phenotype to promote CPSP [19]. GFAP and IBA1 are positive markers of astrocytes and microglia, respectively [20, 21], and NeuN is a positive marker for neurons [22, 23]. In this study, we found that mechanical pain sensitivity and P-CAV-1 levels in the spinal cord were significantly elevated in SMIR rats after an operation. Intriguingly, P-CAV-1 was costained with GFAP-positive astrocytes, IBA1-positive microglia, and NEUN-positive neurons, suggesting that astrocytes, microglia, and neurons might be the anatomical basis of the CAV-1-mediated regulation of CPSP pain information. They may produce intercell functional coupling through CAV-1, thus leading to CPSP.

Blockage of the gap junction (GJ) leads to cytoskeleton remodeling, weakens the coupling between satellite glial cells and neurons after tissue injury, and inhibits mechanical pain sensitivity [24]. Connexin 43 (CX43) is the main GJ of astrocytes and continuously upregulates CX43 to enhance synaptic transmission in the spinal cord, inducing continuous hypersensitivity of mechanical pain [25–27]. CAV-1 and CX43 colocate in glioblastoma cells [28], and the upregulation of CAV-1 upregulates the expression of CX43 and changes the cytoskeleton [29], suggesting that the CAV-1 overexpression after SMIR-induced surgery may promote the functional coupling between astrocyte, microglia, and neurons through the upregulation of CX43, thus contributing to a significant increase in mechanical pain sensitivity.

The regulatory mechanism of adhesion molecules in astrocytes has been unknown till now [30]. Upregulation of cAMP expression alters the morphology of astrocytes, leading to structural and functional alterations between cells and synapses [31]. The activation of cAMP-EPAC-1 results in a 5–10% reduction in the cross-sectional area of astrocytes and a 30–50% increase in astrocytes within 1 hour, which is enough to affect the function of neurons by changing the extracellular geometry [32]. The elevated expression of CAV-1 induces microglia to form round and amoeba morphologies under pathological conditions [33], regulating the fluidity of astrocyte membranes [34]. Both CAV-1 and water channels are involved in the formation/regression of astrocyte telopod swelling [35]. In this study, we found that the EPAC-1 levels were markedly elevated on the 3rd, 7th, and 14th postoperative days. EPAC-1 and P-CAV-1 were colocalized in GFAP-positive astrocytes of the spinal cord, which laid an anatomical basis for their comodulation of the extracellular geometry and pain information of astrocytes.

To clarify the relationship between EPAC-1 and CAV-1 in CPSP, here, we noticed that after intrathecal injection of CAV-1siRNA, SMIR-induced P-CAV-1 overexpression and nociceptive hypersensitivity reaction were partly reversed, but there was no effect on EPAC-1 expression, indicating that SMIR-induced CAV-1 overexpression promoted CPSP. CAV may be the target of postoperative pain sensitivity response, and EPAC-1 could be the upstream regulatory mechanism of CAV-1. Then CE3F4, an EPAC-1 inhibitor [36, 37], and an agonist, 8-PCPT [38, 39], were selected to further prove the above conclusions. It was found that CE3F4 could reverse SMIR-induced p-CAV-1 overexpression and nociceptive hypersensitivity reactions after using CE3F4, suggesting that SMIR-induced pain sensitivity was not only reversed by CAV-1 siRNA but also by CAV-1 siRNA. Moreover, it was reversed by an EPAC-1 inhibitor, and activation of EPAC-1 could upregulate CAV-1 and promote CPSP. In simulating the effects of CPSP, 8-PCPT showed a significant increase in p-Cav-1 expression in the spinal marrow of normal rats and increased pain sensitivity, similar to the changes in the SMIR model. Therefore, EPAC-1/CAV-1 is an important signaling pathway, which can regulate the geometric structure of astrocytes and promote CPSP.

There are some limitations to our study. First, since the CAV-1 and CX43 have a close regulative relationship in the changes of the cytoskeleton, further studies need to pay special attention to this point. Second, the underlying mechanism of the regulation between CAV-1 and astrocyte polarization has not been investigated in the study.

## 5. Conclusion

**Figure 8:**
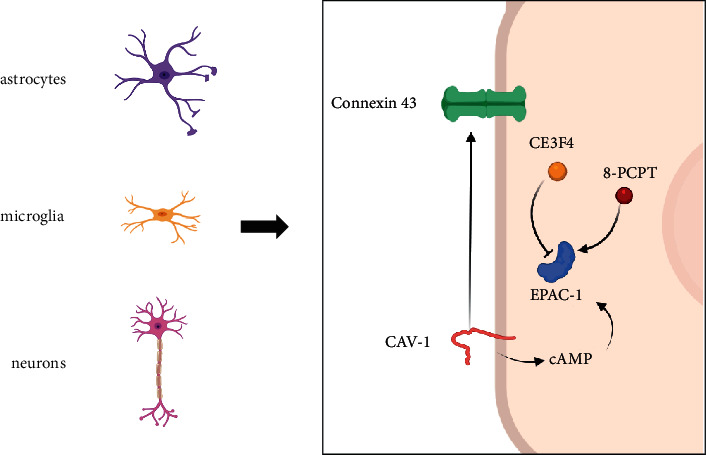
Schematic diagram demonstrating the role of EPAC-1/CAV-1 in CPSP exacerbation. EPAC-1 and CAV-1 were overexpressed after surgery, particularly in astrocytes, microglia, and neurons of the spinal marrow. The overexpression of CAV-1 can induce the upregulation of CX43. And CAV-1 mediates the functional coupling of microglia, astrocytes, and neurons through the cAMP/EPAC-1 pathway. The EPAC-1 inhibitor CE3F4 and the EPAC-1 agonist 8-PCPT can help regulate the expression level of EPAC-1, thus affecting the CPSP exacerbation.

Our study elucidated the presence of EPAC-1 and CAV-1 overexpression after surgery, particularly in spinal marrow astrocytes, microglia, and neurons. Moreover, EPAC-1/CAV-1 plays an important role in CPSP exacerbation. CAV-1 inhibition can partly reverse the SMIR-induced hypersensitivity, but no difference has been found in EPAC-1. Besides, EPAC-1 blockage reversed hypersensitivity and CAV-1 overexpression (Figure 8). Therefore, CAV may be considered as a potential molecular target for CPSP.

## Data Availability

The data used to support the findings of this study are available from the corresponding author upon request.
